# Contribution of Lipids to the Flavor of Mussel (*Mytilus edulis*) Maillard Reaction Products

**DOI:** 10.3390/foods11193015

**Published:** 2022-09-28

**Authors:** Ran Xin, Lixin Ma, Rong Liu, Xuhui Huang, Baoshang Fu, Xiuping Dong, Lei Qin

**Affiliations:** National Engineering Research Center of Seafood, Collaborative Innovation Center of Seafood Deep Processing, School of Food Science and Technology, Dalian Polytechnic University, Dalian 116034, China

**Keywords:** *Mytilus edulis*, polar lipids, nonpolar lipids, Maillard reaction products, flavor

## Abstract

Lipid oxidation and the Maillard reaction are two of the most important reactions affecting the flavor of foods that have been heat-processed. To investigate the contribution of lipids to the mussel’s flavor, the mussel’s Maillard reaction products (MRPs) were prepared with polar lipids (mainly phospholipids) and nonpolar lipids (mainly glycerides), respectively. The effects of polar and nonpolar lipids on the flavor of the MRPs were investigated by sensory evaluation, electronic tongue, electronic nose, ultra-performance liquid chromatography-mass-spectrometry (UPLC-MS) and gas chromatography-mass-spectrometry (GC-MS). From the sensory evaluation results, the polar lipid MRPs had the highest scores. The tastes of polar lipid MRPs and nonpolar lipid MRPs were mainly umami, saltiness and sourness, and there were significant differences in their sour tastes. The flavor compounds in the MRPs were mainly inorganic sulfides, organic sulfides and nitrogen oxides. The odor of polar lipid MRPs was stronger than that of nonpolar lipid MRPs, and the seafood flavor was more obvious. A total of 37 volatile compounds were detected by GC-MS, mainly aldehydes, alcohols and ketones. The addition of polar lipids helped the MRPs to produce more volatile compounds. A total of 177 non-volatile compounds (including amino acids and their derivatives and oligopeptides, etc.) were detected in the samples using UPLC-MS. The non-volatile compounds contained in the no-lipid MRPs, polar lipid MRPs and nonpolar lipid MRPs were significantly different. This study provides a theoretical basis and technical support for the production of mussel MRPs.

## 1. Introduction

Mussel (*Mytilus edulis*) is a bivalve mollusk found along the Bohai Sea, the Yellow Sea, and the East China Sea. The mussel has high nutritional value, and is rich in protein, fat, and essential amino acids [1]. Mussels contain eight essential amino acids, such as valine and leucine, in quantities much higher than those contained in eggs, chickens, ducks, fish and shrimp [2]. Previous studies have shown that the mussel extract has free-radical-scavenging activities and anti-oxidant properties [3]. The glycogen contained in mussels can relieve fatigue, enhance immunity, protect the liver, and promote heart and blood circulation [4]. Mussels contain manganese, zinc, calcium, magnesium, phosphorus, iron and selenium and other inorganic elements and vitamins required by the human body, which can promote blood circulation [5]. With improved living standards, awareness of physical health, and increasing attention to food nutrition and health, mussels are becoming more popular among consumers.

China is the number one producer of mussels globally, producing over 600,000 tonnes per year [6]. In China, mussels are mainly sold fresh, and the utilization of mussels for further processing is still relatively low. In addition to fresh sales, some mussel meat will be processed into convenient or frozen food for sale, some will be made into dried products to extend its shelf life to facilitate transportation, and some even be directly used as feed or abandoned, resulting in waste of resources [7]. Therefore, enhancing the value of the deep processing of mussels is essential. Currently, the industrialization of mussels is still at the stage of small-scale experimental research, which restricts the development of the mussel industry and lowers the actual market value of mussels. Therefore, to increase the economic value of mussels and make full use of marine resources, more research efforts should be invested into research to promote their further processing and industrialization.

The Maillard reaction is a non-enzymatic browning reaction between amino-containing compounds and carbonyl compounds [8]. It plays a crucial role in food processing and storage and generates a wide range of Maillard reaction products (MRPs) [9]. Temperature is an important influencing factor of the Maillard reaction. Excessive temperature may produce some toxic products, such as ketone aldehyde, glyoxal, methylglyoxal, 3-deoxyglucosone (3-DG), heterocyclic amine and acrylamide [10]. However, the Maillard reaction under suitable conditions is beneficial for food processing, and the generated Maillard reaction products contribute to the flavor of food. The flavor of MRPs mainly includes volatile and non-volatile flavors. Volatile flavors mainly refer to odors that people can smell. The volatile flavor is considered the most important determinant of food flavor. The contribution of each volatile compound to flavor depends on its particular odor threshold and concentration. Volatile compounds in aquatic products mainly include aldehydes, alcohols, ketones, aromatic hydrocarbons and sulfur compounds, which are derived from microbial activities, enzymatic reactions and lipid oxidation [11]. Non-volatile flavor mainly refers to water-soluble flavor compounds, which can be perceived by taste. Non-volatile flavor compounds include nitrogenous compounds and non-nitrogenous compounds. Nitrogenous compounds mainly include free amino acids, peptides, organic bases, and nucleotides and their derivatives. Non-nitrogenous compounds mainly include organic acids, sugars, inorganic compounds and other compounds (vitamins, pigments, minerals, etc.) [12].

Seafood is an important source of nutrients in human diets around the world and contains valuable lipids [13]. Lipid oxidation is the main way to produce characteristic flavor, and the characteristic aroma of meat comes from oxidative lipid degradation. In general, the oxidative decomposition of lipids produces a large number of volatile flavor compounds, and the chemical reactions involved mainly include the oxidation and degradation of unsaturated and saturated fatty acids. Unsaturated fatty acids are beneficial to human health and can reduce the viscosity of blood sugar [14,15,16]. Mussels are rich in unsaturated fatty acids, especially polyunsaturated fatty acids, such as docosahexaenoic acid and eicosapentaenoic acid. The total amount of these two unsaturated fatty acids in mussels is more than 27%, higher than in most marine fish, which can promote the growth and development of brain cells, and improve brain function and memory [16]. A large number of scientific studies have shown that lipids and their derivatives play an extremely important role in the production of characteristic flavors in meat. For example, Mottram et al. [17] elaborated on the role of lipids in beef flavor in extensive detail. Pearson et al. [18] also found that a characteristic meat flavor will be produced when the animal fat was heated.

In this paper, the MRPs were prepared by Maillard reaction using mussel enzymatic hydrolysate as raw material. During the MRPs preparation, the extracted mussel polar lipid and nonpolar lipid were added to their respective portions, and the flavor changes of the MRPs were compared by analyzing the changes in the sensory evaluation, electronic tongue, electronic nose, volatile components and non-volatile compounds. This study can enhance the further processing of mussels and provide a theoretical basis for the production of mussel heat-reacted MRPs.

## 2. Materials and Methods

### 2.1. Samples and Chemicals

The mussel sample was purchased from the local Changxing market in Dalian and stored in a −80 °C freezer pending further analysis.

Cyclohexanone (>99.5%), methyl tertiary butyl ether (≥99.9%, HPLC gradient grade), and acetone (99.5%) were purchased from Sigma-Aldrich (St. Louis, MO, USA). Methanol (≥99.9%, HPLC gradient grade), acetonitrile (≥99.9%, HPLC gradient grade), formic acid (98%, HPLC gradient grade), isopropyl alcohol (99.9%, HPLC gradient grade), and anhydrous ethanol (>99.9%) were purchased from Merck (Darmstadt, Germany).

### 2.2. Extraction and Detection of Polar and Nonpolar Lipids from Mussel

The polar and nonpolar lipids were extracted from the mussel according to Abeyrathne et al. [19], with slight modifications. Fresh mussel meat was ground and freeze-dried. Mussel freeze-dried powder (20 g) was weighed, and 60 mL ethyl alcohol was added and stirred for 30 min. The supernatant was filtered with a 0.22 μm filter membrane to obtain the crude lipid solution. Polar lipids were precipitated using cold acetone (50 mL). The precipitate and supernatant were dried with nitrogen to obtain the polar lipids and nonpolar lipids for later use.

The mass fraction of polar and nonpolar lipids in mussels was determined by rod thin layer chromatography coupled with a flame ionization detector (TLC-FID). Scanning speed: 30 s/root; hydrogen gas flow rate: 160 mL/min; air flow rate: 2 L/min. The relative mass fractions of polar lipids and non-polar lipids were obtained by the peak area normalization method.

### 2.3. Preparation of MRPs

**Figure 1:**
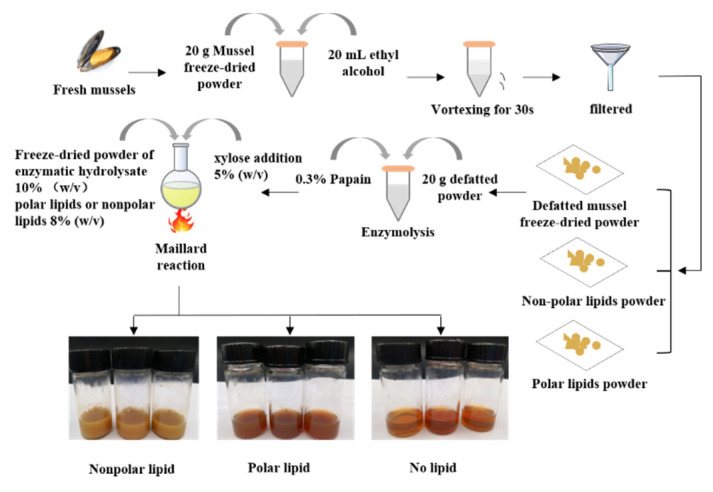
Preparation process of the mussel MRPs.

20 g of defatted mussel freeze-dried powder was dissolved in 1000 mL deionized water 0.3% (*w*/*v*) papain enzyme was added and hydrolyzed at 50 °C for 3 h. The obtained enzymatic hydrolysate was further freeze-dried into powder. The preparation of MRPs was according to the methods of our previous study. We compared the effects of different types of reducing sugar (ribose, glucose, fructose and xylose), reducing sugar additions (1%, 5%, 10%, 15% and 20%, *w*/*v*), reaction times (60, 90, 120 and 150 min) and reaction temperatures (100, 110, 120, 130 and 140 °C) within the Maillard reaction. The results showed that the optimal Maillard reaction condition was a substrate concentration of 10% (*w*/*v*), a reaction temperature of 100 °C, a reaction time of 90 min, and a xylose addition of 5% (*w*/*v*). The freeze-dried powder and polar lipids or nonpolar lipids of 8% (*w*/*v*) were subjected to the Maillard reaction in this condition. Then, the MRPs were dried by spray-drying. The production process of the MRPs in which different polar lipids are added is shown in Figure 1.

### 2.4. Sensory Analysis

The sensory evaluation of MRPs was conducted with reference to the method of Dong et al. [1], with some slight modification. Ten trained sensory panelists carried out sensory analysis, and the samples were scored according to the sensory evaluation criteria in Table S1.

### 2.5. Electronic Nose Analyses

The Electronic Nose Analyses were measured according to the method by Huang et al. [20], with some modifications. A quantity of 5 mL of the enzymatic hydrolysate, no-lipid MRPs, polar lipid MRPs, and nonpolar lipid MRPs were taken in a sample vial (20 mL) and preheated for 30 min at 37 °C in a water bath. The Teflon tube connected with the needle and clean air (400 mL/min) as the carrier gas were used to analyze and detect the odor of the sample. The response data were obtained at 1 s intervals, the measurement time was 50 s, and the time to clean the probe was 40 s. Three sets of parallel experiments were carried out for each set of samples.

### 2.6. Electronic Tongue Analyses

The Electronic Tongue Analyses were measured according to the method by Fan et al. [21], with some modifications. All measurements were performed using the electronic tongue sensor system TS-5000Z (Insent Inc., Fukuoka, Japan). Dilute the enzymatic hydrolysate, no-lipid MRPs, polar lipid MRPs, and nonpolar lipid MRPs with deionized water 50 times, homogenize for 1 min, centrifuge and filter the supernatant. The filtered liquid sample was used for electronic tongue detection. The results were analyzed by TS-5000Z Management Server software.

### 2.7. Volatile Compound Detection

The volatile compounds were measured according to the method by Huang et al. [20] with some modifications. 200 µL each of the enzymatic hydrolysate, no-lipid MRPs, polar lipid MRPs, and nonpolar lipid MRPs were taken into a headspace sample vial (20 mL). The internal standard deuterated cyclohexanone (50 mg/L, 10 µL) was added, and the samples were incubated at 40 °C for 20 min. The manual injection needle extraction time was 40 min. Agilent 7890B GC/7010B GC-MS was used for analysis. Chromatographic conditions: separations were performed using an HP-5MS capillary column (30 m × 250 µm × 0.25 µm); The warming procedure was carried out as follows: the initial column temperature of 45 °C was held for 3 min and increased by 5 °C/min to 280 °C, the temperature was then held there for 10 min. The carrier gas flow rate: 1 mL/min; Injection port temperature: 260 °C. MS/MS conditions: ionization mode: EI; Electron energy: 70 eV; The mass scan range was 35–400; The solvent delay was set to 3.5 min. All assays were performed in triplicate.

### 2.8. Extraction and Detection of Non-Volatile Compounds

100 µL each of the enzymatic hydrolysate, no-lipid MRPs, polar lipid MRPs, and nonpolar lipid MRPs were taken. Subsequently, 250 µL pre-cooled methanol was added, and the samples were shaken for 10 s. At ice temperature, 900 µL of pre-cooled methyl tert-butyl ether (MTBE) was added and vortexed for 2 min. 250 µL of ultrapure water was added. The samples were vortexed for 20 s and incubated at 4 °C for 30 min. The samples were centrifuged at 20,000× *g* for 2 min (4 °C). The lower layer (400 µL) was mixed with 1400 µL of ice-cold Methanol-isopropanol (1:1 *v*/*v*) and vortexed for 2 min. 1500 µL of the supernatant was collected and evaporated by a high-speed vacuum concentrator (Labogene, Scan Speed 40, Lillerød, Denmark). After the sample was reconstituted, 50 µL of the sample was injected into a vial for testing. All assays were performed in triplicate.

The non-volatile compounds were measured according to the method by Chen et al. [22], with some modifications. Ultrahigh performance liquid chromatography (Thermo Scientific, Vanquish, Waltham, MA, USA) coupled with a hybrid quadrupole-orbitrap mass spectrometry (Q Exactive HF-X) was used for method analysis. The separation of target analytes was performed on an Acquity UPLC BEH-C8 column (2.1 × 100 mm; 1.7 μm; Waters Corporation, Milford, MA, USA) equipped with an Acquity BEH-C8 (2.1 × 5 mm; 1.7 μm; Waters Corporation) Van Guard Pre-column. Chromatographic conditions: the mobile phases were 0.1% (*v*/*v*) formic acid/water solution (A) and 0.1% (*v*/*v*) formic acid/acetonitrile solution (B). The following elution gradient was applied: 0–12 min, 5–10%B; 12–42 min, 10–35%B; 42–46 min, 35%B; 46–50 min, 35–50%B; 50–54 min, 50–95%B; 54–62 min, 95%B; 62–64 min, 95–5%B; 64–72 min, 5%B. The flow rate was 0.4 mL/min. The injected volume was 1 µL. The column compartment temperature was kept at 40 °C. MS/MS conditions: the Q Exactive HF-X mass spectrometer was operated under an ESI positive mode for all detections. The full mass scan range was 150–2250 *m*/*z*. Sheath gas flow rate: 60 psi; aux gas flow rate: 25 psi; sweep gas flow rate: 2 psi; spray voltage: 3.60 kV; aux gas heater temp: 370 °C; capillary temp: 380 °C. To monitor the stability of the Q Exactive HF-X mass spectrometer and obtain rich data information, 20 μL aliquots of each sample were prepared for a quality control (QC) sample.

### 2.9. Data Processing

Targeted data acquired on the Q Exactive HF-X were analyzed by MS-DIAL (MS-DIAL program ver. 2.94). Statistical analysis was performed by SPSS 22.0 (SPSS Inc., Chicago, IL, USA). Heatmap, principal components analysis (PCA) and partial least squares discrimination analysis (PLS-DA) were conducted by MetaboAnalyst 4.0 [23]. Radar charts were drawn by Origin 8.0.

## 3. Results and Discussion

### 3.1. Determination of the Mass Fractions of Polar and Nonpolar Lipids by TLC-FID

Table S2 displays the mass fractions of polar and non-polar lipids extracted from mussels determined by TLC-FID. It can be seen that the mass fraction of extracted polar lipids was higher than that of non-polar lipids, which indicates that the extraction effect of polar lipids was better than that of non-polar lipids. In general, the extraction effects of polar lipids and non-polar lipids were strong. It has been reported [24] that the lipid composition of mussels is affected by seasonal changes, and that the total lipid content was higher in summer than in winter. Glycerides and phospholipids are the main components in summer and winter, respectively. Glycerides can provide energy, and phospholipids are the main components of cell membranes.

### 3.2. Sensory Evaluation Results

**Figure 2:**
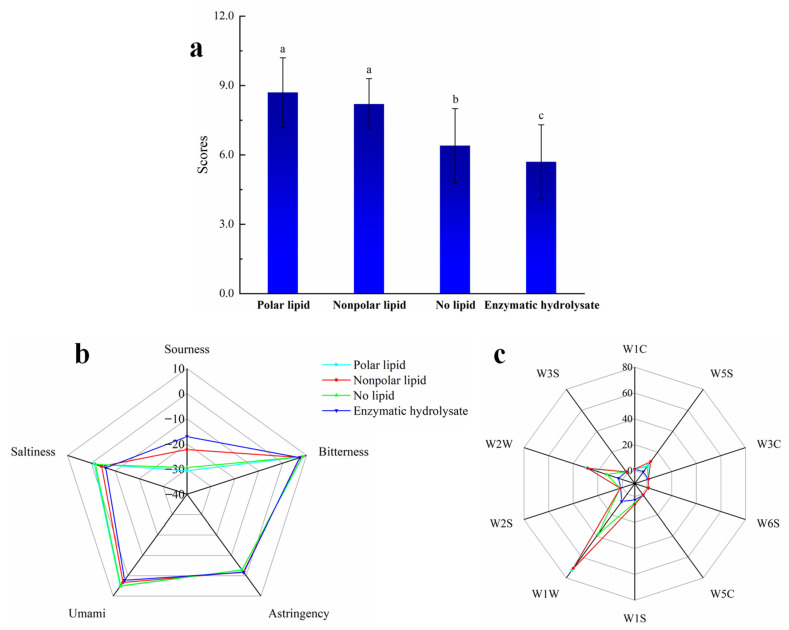
The sensory evaluation, electronic tongue and electronic nose analysis results of MRPs added with different polar lipids: (**a**) the sensory rating bar chart; (**b**) electronic tongue radar map; (**c**) electronic nose radar map. Note: W1C: Benzenes, W5S: Nitrogen oxides, W3C: Ammonias, W6S: Hydrides, W5C: Short-chain alkanes, W1S: Methyls, W1W: Inorganic sulfides, W2S: Alcohols, aldehydes and ketones, W2W: Organosulfur compounds, W3S: Long-chain alkanes.

The process of sensory evaluation, the electronic tongue and the electronic nose were used to measure the enzymatic hydrolysate, no-lipid MRPs, polar lipid MRPs, and nonpolar lipid MRPs to explore the changes and differences in their flavor. The results are shown in Figure 2.

Figure 2a shows that the enzymatic hydrolysate had the lowest score, which indicates that the MRPs had a characteristic odor note that could significantly increase the sensory acceptance of the product. The no-lipid MRPs showed a lower score than polar lipid MRPs and nonpolar lipid MRPs, which was consistent with the previous results, indicating that lipid oxidation can impart a characteristic aroma to food [25]. The sensory scores of the polar lipid MRPs and the nonpolar lipid MRPs were similar, and the polar lipids MRPs had the highest scores. Figure 2b is the radar map of the electronic tongue detection results. From Figure 2b, it can be seen that the umami, saltiness and sourness of the MRPs were changed significantly after adding lipids. The saltiness and umami tastes had the lowest scores in the enzymatic hydrolysate, while scores slightly increased after adding nonpolar lipids, and the highest scores were attained in the no-lipid MRPs and polar lipid MRPs, which indicated that the Maillard reaction had an umami-enhancing effect. The addition of lipids reduced the sourness of the MRPs, while the sourness of the nonpolar lipid MRPs was higher than that of the polar lipid MRPs. The bitterness score was almost unchanged before and after heating, and before and after adding lipid, indicating that the Maillard reaction did not produce bitterness, and the Maillard reaction conditions were suitable. From the results of the electronic tongue, it can be concluded that the rich seafood tastes of MRPs were mainly formed by umami, saltiness, and sourness. Figure 2c is the radar map of the electronic nose detection results. As shown in the radar charts in Figure 2c, the flavor compounds in the MRPs were mainly inorganic sulfides, organic sulfides and nitrogen oxides. The sulfur-containing compounds produced by the Maillard reaction typically had a meaty flavor, so the Maillard reaction has been widely employed in the production of MRPs. The odor substances in the polar lipid or nonpolar lipid MRPs were significantly more than in no-lipid MRPs. This indicates that lipid oxidation plays an important role in the generation of characteristic flavors of the Maillard reaction. The smell of the polar lipid MRPs was stronger than that of the nonpolar lipid MRPs, and the seafood flavor was more obvious.

### 3.3. Determination of Volatile Compounds

A gas chromatography-mass spectrometer (GC-MS) was used to further investigate the content and differences of volatile compounds in enzymatic hydrolysate, no-lipid MRPs, polar lipid MRPs and nonpolar lipid MRPs. The determination results of the volatile compounds in the four samples are shown in Table S3. A total of 37 volatile compounds were detected by GC-MS, including ten alcohols, four acids, eight aldehydes, one aromatic compound, one pyridine, two furans, one thiazole, nine ketones, etc. The most abundant compounds were aldehydes, alcohols and ketones. The content of volatile compounds detected in polar lipid MRPs was the highest, with a total amount of 1029.13 mg/L, and the total content of volatile compounds in nonpolar lipid MRPs was 787.16 mg/L. According to Table S3, the content of aldehydes in the no-lipid MRPs was the highest (169.24 mg/L), and the content became lower with the addition of lipids [26]. According to the study, typical fatty aldehydes such as hexanal, heptanal, and (E)-2-Decenal have an effect on the Maillard reaction, but the effects of different fatty aldehydes on the promotion of the Maillard reaction varies greatly. Both heptanal and hexanal aldehydes have strong fatty aromas, while, after dilution, they have aromas similar to roses and oranges [27]. In addition, alcohols (621.80 mg/L) and ketones (696.79 mg/L) were the most abundant in polar lipid MRPs. Among them, alcohols mainly include 1-often-3-ol (271.37 mg/L) and 1-heptanol (144.13 mg/L). The alcohols have a low threshold and have mushroom or metallic odors [28]. The 1-octen-3-ol is produced by the hydroperoxide of linoleic acid, which has strong grass, mushroom, and fatty odors [28], and is commonly found in aquatic products [29]. Studies have shown that the 1-octen-3-ol is highly correlated with the degree of fat oxidation [30]. The main ketone compound contained in the polar lipid MRPs was 4-methyl-3-Penten-2-one (418.11 mg/L). The main contribution of ketones to the flavor of aquatic products is the aroma of flowers and fruits [31].

**Figure 3:**
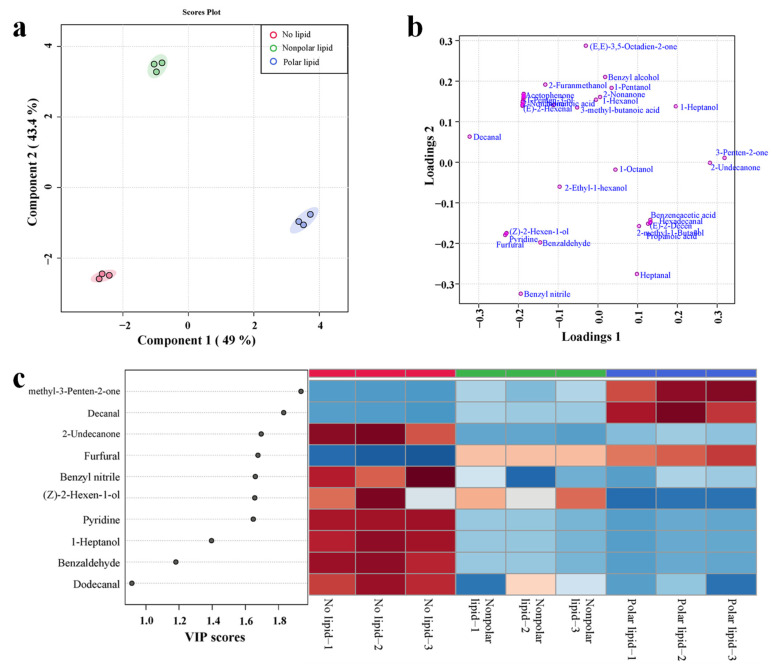
PLS-DA analysis diagram of volatile compounds in MRPs with different polarity lipids: (**a**) scores plot; (**b**) loading plot; (**c**) VIP plot and heatmap.

To further investigate the effect of added lipids on the volatile compounds of MRPs, PLS-DA analysis was performed on the volatile compounds detected in no-lipid, polar lipid and nonpolar lipid MRPs. The PLS-DA analysis plots were shown in Figure 3. The score plot is shown in Figure 3a. The first two principal components accounted for 49.0% and 43.3%, respectively. The cumulative contribution rate was 92.4%. From the score plot, it can be seen that no-lipid MRPs, polar lipid MRPs and nonpolar lipid MRPs are clearly divided into three groups. Nonpolar lipid MRPs are in the second quadrant, no-lipid MRPs are in the third quadrant, and polar lipid MRPs are in the first quadrant; there were significant differences in volatile compounds among the three groups of samples. The loading plots (Figure 3b) show the distribution of volatile compounds in three MRPs. The nonpolar lipid MRPs mainly contained decanal, 2-pentyl-furan, (E,E)-3,5-octadien-2-one, benzyl alcohol, 1-pentanol, 3-methyl-butanoic acid, etc. Decanal had sweet, citrus, floral and waxy aromas, while 2-pentyl-furan and (E,E)-3,5-octadien-2-one had floral and sweet aromas [32]. The main volatile substances contained in polar lipid MRPs were heptanal, benzeneacetic acid, 2-undecanone, 4-methyl-3-Penten-2-one, etc. In particular, heptanal had a fragrant, sweet flavor [32]. Most of the ketones were the products of thermal oxidative degradation of polyunsaturated fatty acids [33]; they usually have sweet floral and fruity aromas, and when the carbon chain of ketones increases, they will show more obvious floral aromas. The main volatile substances contained in no-lipid MRPs were benzaldehydes, which generally had the aroma of bitter almonds, cherries and nuts. Figure 3c shows the VIP value plot and heatmap of volatile compounds with a VIP value greater than 1.0. The VIP score of each compound was calculated as a weighted sum of the squared correlations between the PLS-DA components and the original variables. As shown in Figure 3c, among the volatile compounds with VIP > 1.0, most of them were most abundant in the no-lipid MRPs, mainly 2-undecanone, benzyl nitrile, (Z)-2-Hexen-1-ol, pyridine and 1-heptanol, etc. The volatile compounds (VIP > 1.0) found mist extensively in polar lipid extracts were 4-methyl-3-Penten-2-one, decanal and furfural.

### 3.4. Correlation Analysis of Electronic Nose Results and Volatile Compounds in MRPs with Different Polar Lipids

**Figure 4:**
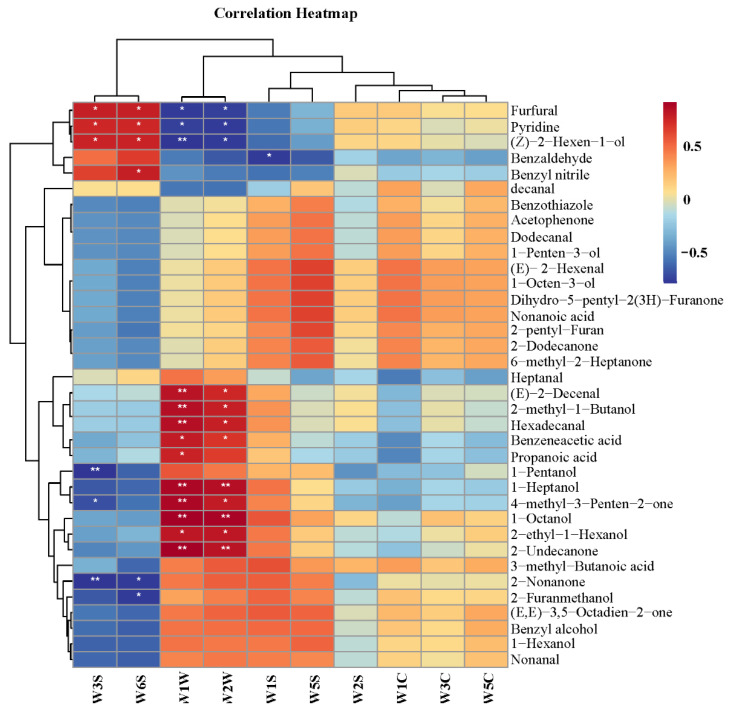
A correlation heatmap of electronic nose results and non-volatile compounds of MRPs with different polar lipids. Note: *: *p* < 0.05; **: *p* < 0.01.

The volatile compounds detected in the MRPs and the electronic nose results were correlated to investigate the contribution and influence of each volatile compound on the odor of the MRPs. The correlation heatmap is shown in Figure 4. Figure 4 shows that the compounds significantly positively correlated with the W1W flavor of the MRPs were (E)-2-Decenal, 2-methyl-1-butanol, hexadecanol, 1-heptanol, 4-methyl-3-penten-2-one, 1-octanol and 2-undecanone. Among them, most of them were alcohol compounds. The compounds that were significantly positively correlated with the W2W flavor of the MRPs were 1-heptanol, 1-octanol and 2-undecanone. From the results of the electronic nose, it ca be seen that W1W and W2W were important odors of the MRPs. Therefore, according to the correlation analysis results, 1-heptanol, 1-octanol and 2-undecanone played a very important role in the flavor of the MRPs.

### 3.5. Determination of Non-Volatile Compounds

**Figure 5:**
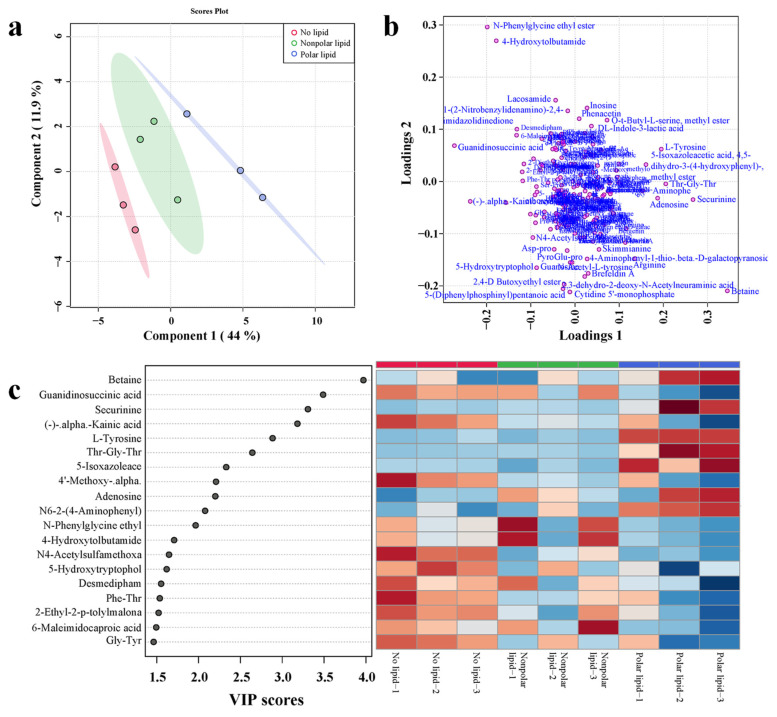
PLS-DA analysis diagram of non-volatile compounds in MRPs with different polarity lipids: (**a**) scores plot; (**b**) loading plot; (**c**) VIP plot and heatmap.

To further investigate the effect of added lipids on the taste of MRPs, PLS-DA analysis was performed on non-volatile compounds detected in no-lipid, polar lipid and nonpolar lipid MRPs. The PLS-DA analysis plots are shown in Figure 5. A total of 177 non-volatile compounds were detected in the samples, mainly including amino acids and their derivatives and oligopeptides, etc. As shown in the score plot (Figure 5a), the first two principal components accounted for 44% and 11.9%, respectively. The cumulative contribution rate was 55.9%. The score plot shows that the non-volatile compounds contained in the no-lipid MRPs, polar lipid MRPs and nonpolar lipid MRPs were significantly different. The loading plot (Figure 5b) presents information on the distribution of nonvolatile compounds in samples. Figure 5c shows the VIP value plot and heatmap of 19 non-volatile compounds with a VIP value greater than 1.5. It can be observed that the content of non-volatile compounds with VIP values greater than 1.5 in the three MRPs samples was significantly different. Non-volatile compounds with higher content in no-lipid MRPs were guanidinosuccinic acid, (-)-alpha-kainic acid, 4′-methoxy-alpha-naphthoflavone, N4-acetylsulfamethoxazole, 5-hydroxytryptophol, desmedipham, Phe-Thr, 2-ethyl-2-p-tolylmalonamide and Gly-Thr. The polar lipid MRPs had the highest amount of betaine, securinine, L-tyrosine, Thr-Gly-Thr, 4,5-dihydro-3-(4-hydroxyphenyl)-5-isoxazoleacetic acid, adenosine and N6-2-(4-aminophenyl)-ethyladenosine. N-phenylglycine ethyl ester, 4-hydroxytolbutamide and 6-maleimidocaproic acid were found in higher content in the nonpolar lipid MRPs.

### 3.6. Analysis of Amino Acids and Their Derivatives

Amino acids and their derivatives are an important class of non-volatile compounds. The 18 amino acids and their derivatives in the non-volatile compounds detected in the enzymatic hydrolyzate, no-lipid MRPs, polar lipid MRPs and nonpolar lipid MRPs are listed in Table S4. It can be seen from Table S4 that the total content of 18 amino acids and their derivatives was highest in the enzymatic hydrolyzate., with content of 1558.55 mg/100 mL. The total contents of amino acids and their derivatives in the enzymatic hydrolysate were reduced after the Maillard reaction was used to prepare the MRPs, and the total content of amino acids and their derivatives in no-lipid MRPs was the lowest at 557.76 mg/100 mL. The total content of amino acids and their derivatives in polar lipid MRPs (1073.32 mg/100 mL) and nonpolar lipid MRPs (624.78 mg/100 mL) was higher than in no-lipid MRPs. It can be seen from the results that the Maillard reaction promotes the participation of amino acids and their derivatives in the reaction, so the content of amino acids and their derivatives in the MRPs was lower than that in the enzymatic hydrolysate. However, the content of amino acids and their derivatives in polar and nonpolar lipid MRPs was higher than than in no-lipid MRPs, probably due to the reaction between lipids and some dicarbonyl compounds in the Maillard reaction. Among the 18 amino acids and their derivatives detected, betaine and O-t-butyl-L-serine methyl ester were found in higher measures. Betaine is a natural compound that exists widely in nature [34]. O-t-butyl-L-serine methyl ester may be a metabolite of serine, which is also a sweet amino acid. Both betaine and serine contribute to the taste of shellfish [35].

It could also be seen from Table S4 that there were nine amino acids and their derivatives with obvious changes in the four samples, namely, betaine, L-arginine, L-tyrosine, benzyl-L-glutamine methyl ester, guanidinosuccinic acid, lacosamide, L-homocystine, argininosuccinic acid and N.epsilon-acetyl-L-lysine. Compared with the enzymatic hydrolysate, the contents of betaine, benzyl-L-glutamine methyl ester, guanidinosuccinic acid and lacosamide were significantly reduced in MRPs. This indicates that these four compounds were lost during the Maillard reaction. Compared with no-lipid MRPs, the amino acids and their derivatives that significantly increased in the MRPs after the addition of nonpolar lipids were limited to L-arginine, while the amino acids and their derivatives that significantly increased in the MRPs with the addition of polar lipids were composed of betaine, L-arginine and L-tyrosine. Amino acids and their derivatives that were higher in polar lipid MRPs than nonpolar lipid MRPs were betaine, L-arginine, L-tyrosine, L-homocystine and N.epsilon-acetyl-L-lysine. From the results, it is clear that the addition of nonpolar lipids had less effect on amino acids and their derivatives than polar lipids.

### 3.7. Oligopeptide Analysis

Oligopeptides are also an important class of non-volatile taste compounds. Therefore, the oligopeptides detected in enzymatic hydrolysate, no-lipid MRPs, polar lipid MRPs and nonpolar lipid MRPs were further analyzed. It can be seen from Table S5 that a total of 38 peptides were detected, of which the total content in the enzymatic hydrolysate was the highest at 314.98 mg/100 mL, followed by the no-lipid MRPs at 283.79 mg/100 mL. The content of peptides in the polar lipid MRPs and nonpolar lipid MRPs were similar, 275.50 mg/100 mL and 256.00 mg/100 mL, respectively. The addition of polar lipids and nonpolar lipids could encourage peptides to participate in the Maillard reaction, indicating that peptides are important substances involved in the Maillard reaction. The most abundant peptides were mainly Ala-Pro, Gly-Tyr, and Pro-Pro. It was found that the taste of the peptide is related to pH. When the pH is 3.5, the peptide presents a bitter taste; when the pH is 6.5, the peptide presents a sweet taste, and when the pH is 9.5, the peptide presents umami and salty taste [36]. Mee-Ra Rhyu [37] suggested that small-molecule peptides with acidic residues (Glu, Asp) may play a large role in the taste. However, van den Oord [38] experimentally confirmed that some peptides with umami taste previously reported do not have umami taste. However, if the peptide will produce umami in the presence of sodium salt, Lioe H N [39] confirmed that the peptide containing glutamyl residues in soybean sauce has no umami effect. Glutamic acid monomers, aspartic acid, and some sweet amino acids in the presence of sodium salts produce umami. It indicates that the peptide will synergize with other taste substances to produce umami.

**Figure 6:**
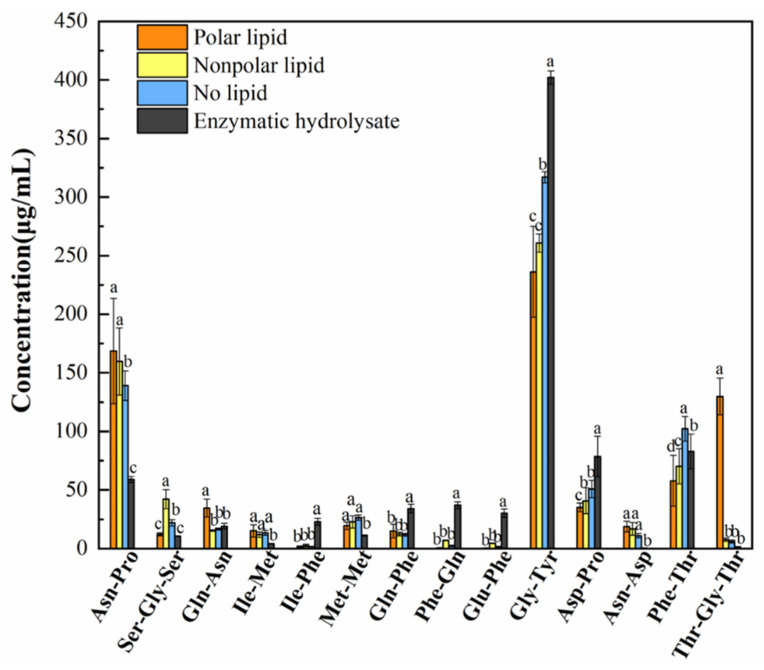
Oligopeptides with obvious changes in MRPs added with different polar lipids.

There were 14 oligopeptides that changed significantly in the enzymatic hydrolysate, no-lipid MRPs, polar lipid MRPs and nonpolar lipid MRPs (Figure 6). Compared with the enzymatic hydrolysate, the contents of Asn-Pro, Ser-Gly-Ser, Gln-Asn, Ile-Met, Met-Met, Asn-Asp and Thr-Gly-Thr increased, while Ile-Phe, Gln-Phe, Phe-Gln, Glu-Phe, Gly-Tyr and Asp-Pro decreased. The contents of Ser-Gly-Ser, Gln-Asn, Asp-Pro, Phe-Thr and Thr-Gly-Thr were significantly different in polar and nonpolar lipid MRPs. The most abundant peptide was Gly-Tyr. It was found that the molecular weight of taste-presenting peptides was mostly less than 3000 u, and the small molecular peptides have strong activity. They could bind to receptors on taste buds to present different tastes [40].

### 3.8. Correlation Analysis of Electronic Tongue Results and Non-Volatile Compounds in MRPs with Different Polar Lipids

**Figure 7:**
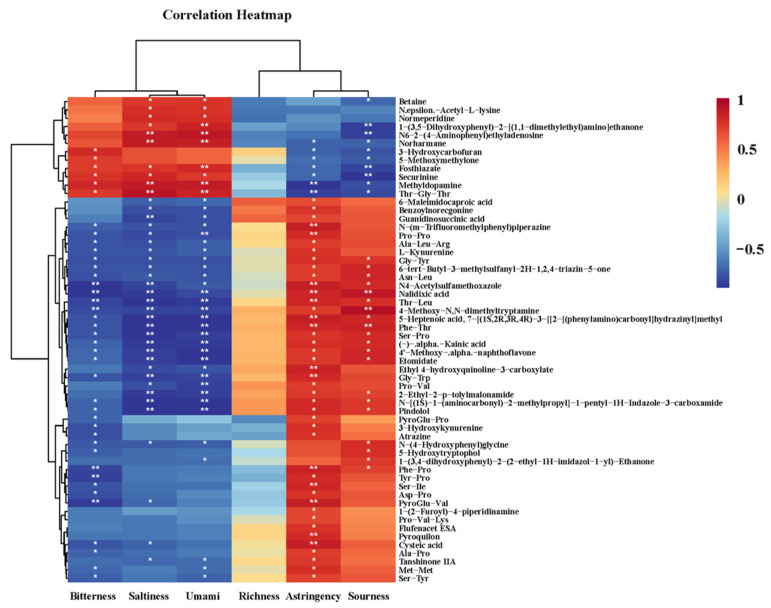
A correlation heatmap of electronic tongue results and non-volatile compounds of mussel MRPs with different polar lipids. Note: *: *p* < 0.05; **: *p* < 0.01.

The non-volatile compounds detected in the MRPs and the electronic tongue results were correlated to investigate the contribution and influence of each non-volatile compound on the taste of the MRPs. The correlation heatmap was shown in Figure 7. Due to a large number of detected non-volatile compounds, this figure only listed 58 non-volatile compounds that were positively correlated with the six tastes of MRPs. From the results of the electronic tongue, it could be concluded that the rich seafood tastes of mussel MRPs were mainly formed by umami, saltiness, and sourness. Therefore, it could be seen from Figure 7 that the compounds that were significantly positively correlated with the saltiness of mussel MRPs were N6-2-(4-aminophenyl)ethyladenosine, normeperidine, Methyldopamine and Thr-Gly-Thr. The compounds that were significantly positively correlated with the umami taste of mussel MRPs included the above four compounds, in addition to two more compounds, 1-(3,5-Dihydroxyphenyl)-2-[(1,1-dimethylethyl) amino] ethanone and Fosthiazate. The compounds that were significantly positively correlated with the acidity of mussel flavor were Nalidixic acid, 4-Methoxy-N,N-dimethyltryptamine and Phe-Thr.

## 4. Conclusions

In this paper, mussel enzymatic hydrolyzate was used as raw material, polar and non-polar lipids were added, and MRPs were prepared by means of the Maillard reaction. It could be seen from the results that the enzymatic hydrolyzate had the lowest sensory evaluation score and the polar lipid MRPs had the highest score. The addition of different polar lipids had a greater effect on the taste of mussel MRPs. The umami, saltiness, and sour tastes of MRPs were significantly changed after lipid addition. The saltiness and umami tastes had the lowest scores in the enzymatic hydrolysate, slightly increased after adding nonpolar lipids, and the highest scores in the no-lipid MRPs and polar lipid MRPs. The acidity of different polar lipid samples was significantly different, and the acidity value of nonpolar lipid MRPs was higher than that of polar lipid MRPs. The flavor compounds in MRPs were mainly inorganic sulfides, organic sulfides and nitrogen oxides. The odor of polar lipid MRPs was stronger than that of nonpolar lipid MRPs, and the seafood flavor was more obvious. A total of thirty-seven volatile compounds were detected in all samples, including ten alcohols, four acids, eight aldehydes, one aromatic compound, one pyridine, two furans, one thiazole, nine ketones, etc. The most abundant com-pounds were aldehydes, alcohols and ketones. The polar lipids MRPs produced more volatile compounds. Alcohols and ketones were the most abundant in the polar lipid MRPs and were much higher than those in the nonpolar lipid MRPs. A total of 177 non-volatile compounds were detected in the samples, mainly including amino acids and their derivatives and oligopeptides, etc. Adding nonpolar lipids had less effect on amino acids and their derivatives than polar lipids. The addition of polar and nonpolar lipids facilitated peptide participation in Maillard reactions. The MRPs prepared from mussel in this study had a strong seafood flavor, which laid a solid foundation for the high value-added processing and utilization of mussels in the future.

## Data Availability

The datasets generated for this study are available on request to the corresponding author.

## Supplementary Materials

The following supporting information can be downloaded at: https://www.mdpi.com/article/10.3390/foods11193015/s1. Table S1: Sensory evaluation descriptors and standards; Table S2: Determination of polar and non-polar lipids in mussels by TLC-FID; Table S3: Table of changes of volatile matter content in MRPs adding different polar lipids (mg/L); Table S4: Table of changes of amino acids and their derivatives in MRPs with different polar lipids added (mg/100 mL); Table S5: Table of changes of oligopeptides in MRPs with different polar lipids added (mg/100 mL).
