# Supplementary material for: Contribution of Lipids to the Flavor of Mussel (Mytilus edulis) Maillard Reaction Products
Source: Foods. 2022 Sep 28;11(19):3015. doi: 10.3390/foods11193015 (PMC9562662; doi:10.3390/foods11193015)
Supplement: Supplementary file 1 [file foods-11-03015-s001.zip › foods-1890382-supplementary.pdf]

Contribution of lipids on the flavor of mussel (*Mytilus edulis*) Maillard Reaction Products

Ran Xin [1], Lixin Ma, Rong Liu, Xuhui Huang, Baoshang Fu, Xiuping Dong, Lei Qin *

[1] National Engineering Research Center of Seafood, Collaborative Innovation Center of Seafood Deep Processing, School of Food Science and Technology, Dalian Polytechnic University, Dalian 116034, China
* Correspondence: qinlei@dlpu.edu.cn; Tel: +86 411 86323262

Table S1. Sensory evaluation descriptors and standards.

Sensory evaluation standards	score
It has the aroma of seafood, and the smell is rich and full. The color is sepia or brown, with a long aftertaste and no burnt smell.	10
Seafood smells prominent, the color is brown or yellow, with a long aftertaste and no burnt smell.	8
A bit of seafood smell, the color is pale-yellow, with a long aftertaste. It has a slightly fishy and bitter taste or a distinctly mushy taste	6
Seafood flavor is mild, the color is pale-yellow, the aftertaste is not long. it has fishy and bitter taste	4
No seafood smell, the color is dark brown. it has a strong burnt or fishy smell.	2
No seafood smell, the color is black. it has a strong burnt or fishy smell.	0

Table S2. Determination of polar and non-polar lipids in mussels by TLC-FID.

Lipid species	Polar lipid	Nonpolar lipid
Mass fraction (%)	92.00±0.50[a]	76.00±0.24[b]

Table S3. Table of changes of volatile matter content in MRPs adding different polar lipids (mg/L).

Number	Retention time (min)	Compound	Polar lipid MRPs	Nonpolar lipid MRPs	No lipid MRPs	Enzymatic hydrolysate
Aldehydes						
1	13.359	Heptanal	15.64±5.66[a]	Nd.	2.69±0.47[b]	Nd.
2	14.707	(E)-2-Hexenal	Nd.	4.36±1.26[a]	3.08±0.53[a]	Nd.
3	23.091	Nonanal	7.16±0.85[c]	16.79±6.81[b]	31.50±5.22[a]	Nd.
4	27.45	Decanal	Nd.	Nd.	129.28±45.87 [a]	Nd.
5	27.463	Benzaldehyde	66.94±15.07[a]	37.47±6.53[b]	Nd.	Nd.
6	31.087	(E)-2-Decenal	14.47±8.94[a]	Nd.	Nd.	Nd.
7	35.133	Dodecanal	Nd.	Nd.	Nd.	Nd.
8	45.526	Hexadecanal	2.97±0.28 [a]	Nd.	2.69±0.47[a]	Nd.
Total amount			107.18±30.73[b]	58.62±14.60[c]	169.24±52.56[a]	-
Alcohols						
9	11.73	1-Penten-3-ol	28.85±6.14[a]	2.42±0.99[b]	Nd.	Nd.
10	14.252	2-methyl-1-Butanol	18.50±0.23[a]	Nd.	Nd.	Nd.
11	21.275	1-Hexanol	Nd.	23.32±3.44[a]	Nd.	Nd.
12	23.258	(Z)-2-Hexene-1-ol	Nd.	Nd.	9.91±0.89[a]	Nd.
13	25.421	1-Octen-3-ol	271.37±25.59[a]	Nd.	Nd.	Nd.
14	25.425	1-Heptanol	144.13±20.94[a]	111.01±21.78[a]	7.81±0.37[a]	Nd.
15	26.615	2-ethyl-1-Hexanol	75.60±19.28[b]	103.11±29.32[a]	61.23±5.08[b]	114.09±9.93[a]

16	28.686	1-Octanol	29.04±11.56[a]	37.31±6.84[a]	15.46±3.40[b]	Nd.
17	31.4	2-Furanmethanol	25.46±9.64[b]	159.19±74.92[a]	15.19±3.71[b]	Nd.
18	36.352	Benzyl alcohol	28.85±6.14[a]	45.36±6.95[a]	Nd.	Nd.
Total amount			621.80±99.52[a]	481.72±144.24[b]	109.60±13.45[c]	114.09±9.93[c]
Acids						
19	28.173	Propanoic acid	17.26±7.47[a]	Nd.	Nd.	Nd.
20	31.688	3-Methyl-butanoic acid	145.96±22.33[a]	114.21±5.86[a]	17.07±6.67[b]	33.06±7.04[b]
21	42.156	Nonanoic acid	Nd.	Nd.	Nd.	Nd.
22	48.795	Benzeneacetic acid	36.05±41.20[a]	Nd.	Nd.	Nd.
Total amount			199.27±70.97[a]	114.21±5.86[a]	17.07±6.67[b]	33.06±7.04[b]
Esters						
23	39.058	Phenol	16.35±6.78[a]	Nd.	Nd.	Nd.
Furan						
24	15.362	2-pentyl-Furan	Nd.	33.48±4.59[a]	Nd.	Nd.
25	25.638	Furfural	Nd.	Nd.	7.05±0.92[a]	Nd.
Pyridine						
26	13.05	Pyridine	Nd.	Nd.	1.70±0.11[a]	Nd.
Ketone						
27	11.121	4-methyl-3-Penten-2-one	418.11±66.25[a]	16.63±7.82[b]	Nd.	Nd.
28	15.542	6-methyl-2-Heptanone	Nd.	12.05±3.55[a]	Nd.	Nd.

29	16.114	1-Pentanol	13.45±0.70[b]	22.33±12.98[a]	Nd.	Nd.
30	22.862	2-Nonanone	35.94±4.83[a]	44.71±4.52[a]	Nd.	Nd.
31	29.016	(E, E)-3,5-Octadien-2-one	5.69±1.34[b]	41.28±7.75[a]	Nd.	Nd.
32	29.885	2-Undecanone	223.60±41.94[a]	14.00±2.07[b]	Nd.	Nd.
33	31.387	Acetophenone	Nd.	37.81±14.31[a]	Nd.	Nd.
34	35.316	2-Dodecanone	Nd.	5.91±0.81[a]	Nd.	Nd.
35	39.563	dihydro-5-pentyl-2(3H)-Furanone	Nd.	Nd.	Nd.	Nd.
Total amount			696.79±115.06[a]	194.72±53.81[b]	-	-
Thiazole						
36	38.01	Benzothiazole	Nd.	4.41±0.85[a]	Nd.	Nd.
Others						
37	37.63	Benzyl nitrile	3.49±0.75[c]	Nd.	47.52±14.13[a]	10.68±0.10[b]
Total			1029.13±323.91[a]	787.16±223.95[b]	352.18±87.84[c]	157.83±17.07[d]

[1] Different superscript letters represent significant difference ($p \leq 0.05$).
[2] Nd: not detected.

Table S4. Table of changes of amino acids and their derivatives in MRPs with different polar lipids added (mg/100mL).

Compound	Retention time	Enzymatic hydrolysate	No lipid MRPs	Polar lipid MRPs	Nonpolar lipid MRPs
Betaine	1.056	373.98±119.55^{a}	174.13±70.80^{b}	369.32±128.67^{a}	167.79±56.73^{b}
N-.alpha-Acetyl-L-ornithine	1.063	29.87±1.31^{a}	28.07±1.07^{a}	31.69±1.39^{a}	26.97±1.47^{a}
L-Arginine	1.113	2.11±0.65^{c}	12.38±1.94^{b}	19.47±8.21^{a}	13.81±5.14^{a}
Glufosinate	1.139	53.33±0.56^{a}	51.28±1.52^{a}	51.62±5.50^{a}	47.96±2.37^{a}
O-t-Butyl-L-serine, methyl ester	1.153	459.34±7.24^{a}	428.47±6.41^{a}	451.34±33.94^{a}	432.35±25.31^{a}
Diethyl L-glutamate	1.19	14.25±1.22^{a}	13.14±0.32^{a}	12.50±1.81^{a}	12.11±0.88^{a}
DL-2-Aminocaprylic acid	1.287	15.20±0.55^{a}	15.37±0.85^{a}	16.88±0.81^{a}	14.73±0.62^{a}
L-Tyrosine	1.364	77.43±3.40^{b}	76.58±6.62^{b}	124.81±6.87^{a}	73.3±4.96^{b}
Benzyl-L-glutamine methyl ester	1.382	7.74±0.56^{a}	0.78±0.16^{b}	0.73±0.15^{b}	1.11±0.31^{b}
Guanidinosuccinic acid	1.808	83.14±3.47^{a}	44.35±3.68^{b}	8.90±6.86^{c}	35.66±19.15^{b}
N-(4-Hydroxyphenyl) glycine	2.05	17.18±1.51^{a}	18.59±0.94^{a}	16.77±0.64^{a}	16.84±0.51^{a}
Cysteic acid	2.57	5.60±0.23^{a}	5.50±0.13^{a}	4.83±0.05^{a}	5.34±0.28^{a}
Lacosamide	2.676	21.07±1.71^{a}	1.16±0.41^{b}	1.05±0.95^{b}	2.36±1.74^{b}
L-Homocystine	3.432	0.14±0.01^{a}	1.97±0.06^{b}	2.58±0.53^{b}	2.01±0.41^{b}
N-Acetyl-L-tyrosine	3.446	12.12±1.57^{a}	12.25±1.43^{a}	10.68±1.44^{a}	11.03±1.02^{a}
Argininosuccinic acid	3.545	0.07±0.04^{b}	1.34±0.26^{a}	1.02±0.17^{a}	1.64±0.39^{a}
Pantothenic acid	3.895	6.37±0.31^{a}	5.96±0.14^{a}	6.06±0.14^{a}	5.31±0.45^{a}
N.epsilon.-Acetyl-L-lysine	3.993	0.09±0.04^{b}	0.88±0.04^{a}	1.09±0.09^{a}	0.92±0.13^{a}

Total amount	1558.22±308.55^{a}	557.76±230.29^{c}	1073.32±326.84^{b}	624.78±209.06^{c}

[1] Different superscript letters represent significant difference ($p \leq 0.05$).

Table S5. Table of changes of oligopeptides in MRPs with different polar lipids added (mg/100 mL).

Compound	Retention time (min)	Enzymatic hydrolysate	No lipid MRPs	Polar lipid MRPs	Nonpolar lipid MRPs
Pro-Val	1.085	11.52±0.48[a]	12.32±0.84[a]	10.56±0.85[a]	9.52±3.45[a]
Ser-Pro	1.093	15.19±0.41[a]	11.28±0.64[a]	8.33±1.75[b]	9.59±0.94[a]
Met-Ala	1.137	4.27±3.30[a]	5.86±0.31[a]	5.84±0.58[a]	5.27±0.13[a]
Thr-Gly-Thr	1.179	0.10±0.07[b]	0.61±0.12[b]	9.75±5.81[a]	0.75±0.16[b]
Ala-Pro	1.198	50.65±3.83[a]	43.85±1.57[b]	39.93±2.38[b]	40.85±2.37[b]
Glu-Val	1.219	5.59±0.67[a]	4.66±0.28[a]	3.73±0.44[a]	4.21±0.24[a]
Ser-Ile	1.282	5.13±0.27[a]	4.37±0.09[a]	3.86±0.44[a]	4.03±0.21[a]
Ala-leu	1.312	6.73±0.08[a]	6.43±0.63[a]	5.72±0.45[a]	5.68±0.22[a]
Asn-Leu	1.314	6.17±0.08[a]	5.51±0.45[a]	4.38±0.42[a]	4.51±0.18[a]
Gly-Tyr	1.317	40.20±0.57[a]	31.69±0.46[b]	23.62±3.88[c]	26.07±0.78[c]
Thr-Leu	1.35	5.97±0.65[a]	5.22±0.35[a]	4.36±0.14[a]	4.53±0.23[a]
Ser-Leu	1.384	3.44±0.22[a]	2.79±0.05[a]	2.75±0.06[a]	2.76±0.13[a]
Gly-Leu	1.385	10.37±0.45[a]	9.16±0.31[a]	8.59±0.32[a]	8.63±0.51[a]
Pro-Pro	1.416	62.65±3.42[a]	56.03±4.14[a]	46.68±3.43[a]	50.15±2.66[a]
Pro-Leu	1.465	11.15±0.58[a]	10.73±0.61[a]	10.30±0.57[a]	10.34±0.83[a]
Glu-Pro	1.524	7.61±1.01[a]	6.47±0.71[a]	5.49±0.65[a]	5.86±0.58[a]
Asp-Pro	1.529	7.85±1.73[a]	5.08±0.73[b]	3.52±0.37[c]	4.08±1.10[b]
Ala-Leu-Arg	1.637	2.81±0.16[a]	2.24±0.26[a]	1.89±0.15[a]	2.08±0.16[a]
Gln-Phe	1.694	3.42±0.37[a]	1.19±0.12[b]	1.49±0.59[b]	0.85±0.69[b]
Asn-Pro	1.735	5.90±0.24[c]	13.91±1.27[b]	16.86±4.49[a]	15.97±2.87[a]
Phe-Gln	2.047	3.70±0.29[a]	0.24±0.06[b]	0.16±0.15[b]	0.48±0.36[b]
Phe-Thr	2.081	8.29±1.48[b]	10.22±1.05[a]	5.78±2.18[d]	7.01±1.50[c]
Gly-Phe	2.386	3.02±0.16[a]	2.71±0.20[a]	2.43±0.18[a]	2.47±0.19[a]
Glu-Ala	2.416	2.69±0.05[a]	2.72±0.12[a]	2.61±0.06[a]	2.57±0.26[a]
Ile-Phe	2.574	2.29±0.29[a]	0.17±0.03[b]	0.12±0.09[b]	0.26±0.12[b]
Ile-Met	2.64	0.43±0.02[b]	1.34±0.20[a]	1.51±0.52[a]	1.19±0.23[a]
Tyr-Pro	3.227	1.87±0.25[a]	1.71±0.13[a]	1.46±0.12[a]	1.50±0.06[a]
Glu-Phe	3.402	3.02±0.36[a]	0.14±0.05[b]	0.04±0.02[b]	0.31±0.25[b]
Ile-Ile	3.434	2.70±0.27[a]	2.45±0.14[a]	2.18±0.25[a]	2.23±0.17[a]
Asn-Asp	3.812	0.05±0.01[b]	1.09±0.19[a]	1.53±0.75[a]	1.68±0.54[a]
Pro-Val-Lys	3.963	1.91±0.11[a]	1.79±0.10[a]	1.49±0.16[a]	1.65±0.15[a]
Gly-Trp	4.000	2.27±0.19[a]	1.93±0.11[a]	1.62±0.09[a]	1.75±0.15[a]
Cys-Val	4.203	7.71±0.81[a]	6.64±0.69[a]	6.46±0.71[a]	6.48±0.46[a]

Ser-Gly-Ser	4.343	1.07±0.03[c]	2.20±0.27[b]	1.94±1.26[c]	3.16±2.0[a]
Gln-Asn	5.807	1.91±0.24[b]	1.67±0.09[b]	3.46±0.76[a]	1.54±0.06[b]
Met-Val	6.642	1.93±0.11[a]	2.89±0.09[a]	3.39±1.59[a]	1.94±1.16[a]
Met-Met	7.032	1.11±0.06[b]	2.63±0.23[a]	1.94±0.33[a]	2.28±0.52[a]
Phe-Pro	9.592	2.29±0.25[a]	1.85±0.06[a]	1.73±0.07[a]	1.77±0.01[a]
Total amount		314.98±23.57[a]	283.79±17.75[a]	257.50±37.06[b]	256.00±26.63[b]

[1] Different superscript letters represent significant difference ($p \leq 0.05$).
